# Host jumps need not be common just because spillover is

**DOI:** 10.1371/journal.pbio.3003682

**Published:** 2026-03-20

**Authors:** Mete Yuksel, Nicole Mideo

**Affiliations:** Department of Ecology and Evolutionary Biology, University of Toronto, Toronto, Canada

## Abstract

Can we predict which pathogen will be responsible for the next pandemic? Emergence risk is a hotly debated topic and a new study in PLOS Biology challenges the idea that pathogens that frequently spill over are more likely to emerge.

Every time a pathogen that normally spreads in wildlife or domestic animals spills over into humans, there is a chance of a pandemic. Will the pathogen successfully sustain transmission, or fail to do so? The answer to this question involves a complicated chain of interactions unfolding at the interface of human and animal populations [1,2].Understanding the ecological and evolutionary factors that make disease emergence more likely, and using this information to enact countermeasures, are key goals of research on infectious diseases (e.g., [3,4]). Since the emergence of SARS-CoV-2 in humans, predicting which pathogens are most risky seems even more urgent; however, available data are often too limited, incomplete, and/or biased to be up to this task [5].

Writing in *PLOS Biology*, Simony and Kennedy [6] point out that despite the complicated biological processes at play after a spillover event, outcomes are binary: success in the new host population or not. Thus, they propose thinking about emergence as the flip of a coin to reason about risk. Pathogens that have more opportunities for spillover – more flips of the coin – seem riskier, but with more opportunities comes more data, and pathogens whose flips keep coming up empty suggest the pathogen may not be that risky after all.

Simony and Kennedy [6] formalize this metaphor and weigh these competing effects using tools from stochastic processes and Bayesian statistics. Their work clarifies what information about the probability of disease emergence is contained in (1) the rate of spillover and (2) the timescale over which a pathogen has been able to spill over (Fig 1). While these two aspects of the emergence process represent different ways of getting more coin flips, their influence on risk is not the same.

**Fig 1:**
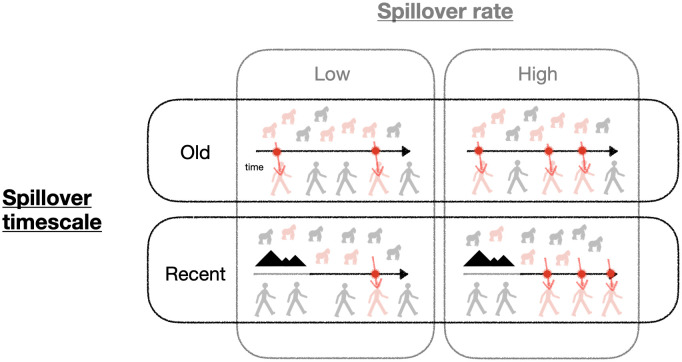
Processes that can potentially influence emergence risk. Simony and Kennedy [6] investigate the role of spillover rate (*λ* in their model) and the timescale over which spillovers have happened (*T*_*p*_ in their model) on the probability that a disease circulating in a reservoir host (here, gorillas) emerges in humans. Red viruses and arrows indicate spillover events; infected individuals (gorillas and humans) are shown in red, and uninfected individuals are shown in gray. Time moves forward according to the black arrows, which are shown in gray when spillover is not possible, e.g., due to geographic barriers. Their results show the most informative comparison for adjudicating relative emergence risk, as indicated by the dark boxes, is spillover timescale: pathogens that recently began spilling over into humans (“recent” associations) are riskier than ones that have been spilling over for a long time (“old” associations). In contrast, little can be learned about emergence risk from comparing spillover rates (light boxes).

The analysis of Simony and Kennedy [6] shows, counterintuitively, pathogens that spillover at higher rates are not inherently more risky than those spilling over at lower rates. To illustrate this point, they explore three possible scenarios for what is believed in advance about the capacity of the pathogen to sustain transmission in humans after a spillover event (the prior, in Bayesian parlance), conditioning on the fact that the pathogen has not yet emerged. Emergence risk does increase with spillover rate (albeit with diminishing returns) in the case of a heavily right-skewed prior, which assumes the vast majority of pathogens sampled during spillover events are unable to successfully sustain transmission in humans. For the other priors, emergence risk is maximized at low or intermediate spillover rates. These include a U-shaped distribution, which assumes that pathogens are either very likely or very unlikely to successfully establish after a spillover event, and a bimodal distribution, which assumes most pathogens are unlikely to sustain transmission in the new host but a small fraction are capable. While Simony and Kennedy [6] point out that specifying an appropriate prior is non-trivial, in our minds, these latter two distributions could reasonably describe a pathogen that is well adapted to its reservoir host, but requires one or a few mutations to transmit well in humans (e.g., [7]). In any case, this sensitivity to the prior demonstrates that there is little information in the spillover rate on its own for predicting emergence risk.

On the other hand, the timescale over which a pathogen has been able to spill over (what the authors call the “novelty” of the pathogen) is a much more reliable predictor of emergence risk, across prior distributions. Put simply, if a pathogen has been spilling over for ages and has not yet emerged, this is strong evidence against emergence in the future. By the same logic, less comfort can be taken from failures to emerge of a pathogen that only recently gained opportunities to spill over (e.g., through new interactions between humans and the pathogen’s reservoir host). Quantifying the duration of time over which a pathogen has been able to spill over may therefore be most useful in adjudicating relative emergence risk.

It is interesting to think about the results of Simony and Kennedy [6] in light of pathogens for which there is ongoing concern about emergence. For example, avian influenza A viruses (e.g., H5N1, one of several subtypes referred to as “bird flu”) instil fear of a pandemic in many. Part of that fear is due to high spillover rates. One source of these viruses is live bird markets, and a study of respiratory samples from market workers in 2017 revealed high levels of exposure [8]. Is it only a matter of time before evolution finds a way? Simony and Kennedy [6] show that, a priori, we should be no more scared of H5N1 than something that spills over much less frequently.

The H5N1 lineage circulating in live bird markets in 2017 was clade 2.3.2.1a [8], but the lineage causing massive outbreaks in wild birds, poultry and various mammal species is a sister clade, 2.3.4.4b [9]. The analysis of Simony and Kennedy [6] would suggest that, all else equal, we should be more scared of 2.3.4.4b since it has been circulating, with opportunities for spillover, for less time. Herein lies one challenge with trying to apply their analysis to a particular pathogen (which, we emphasize, was not their goal): to what extent does a new lineage represent a novel pathogen (a past spillover timescale of zero years, in the formalism of the paper)? Given a shared common ancestor, does the failure of 2.3.2.1a to emerge over the last decade give us confidence that 2.3.4.4b will also fail? Or are these sufficiently different viruses that our past history with 2.3.2.1a is irrelevant?

Assessing novelty may be straightforward when the breakdown of geographic barriers leads to new associations, but seems a more challenging task in the face of pathogen evolution. Simony and Kennedy [6] contend – and we agree – that evolution is unlikely to make past history completely irrelevant, but how informative it is may boil down to the particular biology of the system (e.g., if there has been a fundamental shift of emergence-related traits that is not already captured in prior beliefs about the likelihood of sustained transmission). Regardless, the thought-provoking analysis of Simony and Kennedy [6] challenges a tantalizingly intuitive view that the pathogens that spillover frequently are of most concern for emergence because they get the most flips of the coin. Instead, their model suggests that an understanding of how long those flips have been coming up empty holds the key to predicting and curtailing the next pandemic before it arrives.
